# Strigolactone Signaling Genes Showing Differential Expression Patterns in Arabidopsis *max* Mutants

**DOI:** 10.3390/plants8090352

**Published:** 2019-09-19

**Authors:** Manu Kumar, Inyoung Kim, Yeon-Ki Kim, Jae Bok Heo, Mi Chung Suh, Hyun Uk Kim

**Affiliations:** 1 Department of Bioindustry and Bioresource Engineering, Plant Engineering Research Institute, Sejong University, Seoul 05006, Korea; manukumar@sejong.ac.kr (M.K.); kiy88410@gmail.com (I.K.); 2 Department of Biosciences and Bioinformatics, Myongji University, Yongin 17058, Korea; kim750a11@mju.ac.kr; 3 Department of Molecular Genetic Biotechnology, Dong-A University, Busan 49315, Korea; jbheo72@dau.ac.kr; 4 Department of Life Sciences, Sogang University, Seoul 04107, Korea; mcsuh@sogang.ac.kr

**Keywords:** strigolactone, MAX, biosynthesis, signaling, branching, microarray

## Abstract

Strigolactone (SL) is a recently discovered class of phytohormone that inhibits shoot branching. The molecular mechanism underlying SL biosynthesis, perception, and signal transduction is vital to the plant branching phenotype. Some aspects of their biosynthesis, perception, and signaling include the role of four *MORE AXILLARY GROWTH* genes, *MAX3, MAX4, MAX1*, and *MAX2*. It is important to identify downstream genes that are involved in SL signaling. To achieve this, we studied the genomic aspects of the strigolactone biosynthesis pathway using microarray analysis of four *max* mutants. We identified SL signaling candidate genes that showed differential expression patterns in *max* mutants. More specifically, *1-AMINOCYCLOPROPANE-1-CARBOXYLATE SYNTHASE 4* (*ACC4*) and *PROTEIN KINASE 3* (*PKS3*) displayed contrasting expression patterns, indicating a regulatory mechanism in SL signaling pathway to control different phenotypes apart from branching phenotype.

## 1. Introduction

Strigolactones (SLs) are known for stimulating seed germination in species of the genera *Orobanche*, *Phelipanche*, and *Striga* [1,2,3]. Thus, the release of SLs from roots into the rhizosphere under nutrient-deprived conditions is the first response for the symbiotic association with host arbuscular mycorrhizal fungi to boost its capacity to uptake sugars, minerals, and water [1,4]. This process validates the importance of SLs in root exudates as a chemical signal that initiates germination and induces branching to promote the pre-symbiotic growth of these fungi for access to phosphorus, water, and other minerals, and in return provides photosynthetic products [1,5,6,7]. During phosphate starvation, plants release a large amount of SLs [8,9].

Recently, the discovery of several high branching mutants in Arabidopsis, rice, and pea revealed SL’s role as a carotenoid-derived plant hormone that predominantly regulates axillary shoot branching in an auxin-dependent manner [10,11]. In *Arabidopsis thaliana*, pea, tomato, and petunia, SLs are reportedly involved in other developmental processes, such as lateral root formation, primary root growth, root hair elongation, secondary growth in the stem, senescence, adventitious root formation, and determining root architecture by inhibiting the growth of primary roots [12,13,14,15,16,17,18,19,20]. According to recently published reports, SLs are also involved in the plant response to abiotic stress [21,22,23,24,25,26].

SLs as derivatives of carotenoids and can be divided into two groups: oxygen-free carotene (for example, β-carotene) and oxygen-containing xanthophyll (for example, lutein) [27,28,29]. Carotenoids differ in their isomeric states by their double bond; be they *cis*- or *trans*-configured carotenoids. The plant can synthesize and accumulate both forms as an intermediate of the precursor of a pathway (for example, SLs) [25,28,30]. Oxidative cleavage of a double bond in the isomeric form of carotenoids initiates the synthesis of SLs [31]. 

Genes involved in the SL signaling pathway were identified once it was known that a deficiency of SLs leads to more a branching phenotype in pea, *Arabidopsis* and, rice mutant [22,25]. In *Arabidopsis thaliana*, carotenoid dioxygenases *MORE AXILLARY GROWTH3* (*MAX3*; *AtCCD7*), *MAX4* (*AtCCD8*), and cytochrome P450 *MAX1* (*AtCyp711A1*) are reported to be involved in the biosynthesis of branch inhibiting signal [16,19,32,33,34,35,36,37], whereas the F-box and Leu-rich repeats containing MAX2 protein probably act either in signal transduction or perception [35,37,38]. All *max* mutants display increased numbers of shoot branches, and this mutant phenotype can be rescued in a *MAX2*-dependent manner by the external application of the synthetic strigolactone analog GR24 [10,11,19].

In this study, we investigated downstream genes that responded or regulated the strigolactone pathway using a microarray in four Arabidopsis *max* mutants; *max3, max4, max1, and max2.* We found that 34.1% of the total genes were differentially expressed; among them, 18.7% were up-regulated, and 15.4% were down-regulated. Most of the genes are involved in different development processes, such as epidermal cell differentiation, xyloglucan metabolic process, dormancy process, response to stimulus, and nucleobase transport. Expression analysis of *max* mutants also revealed plant development-related downstream genes (*ACC4*, *PKS3*, *LRX1*, and *LAX1*) in SL signaling.

## 2. Results and Discussion

### 2.1. The Branched Phenotype of max Mutants and Strigolactone Signaling

**Figure 1:**
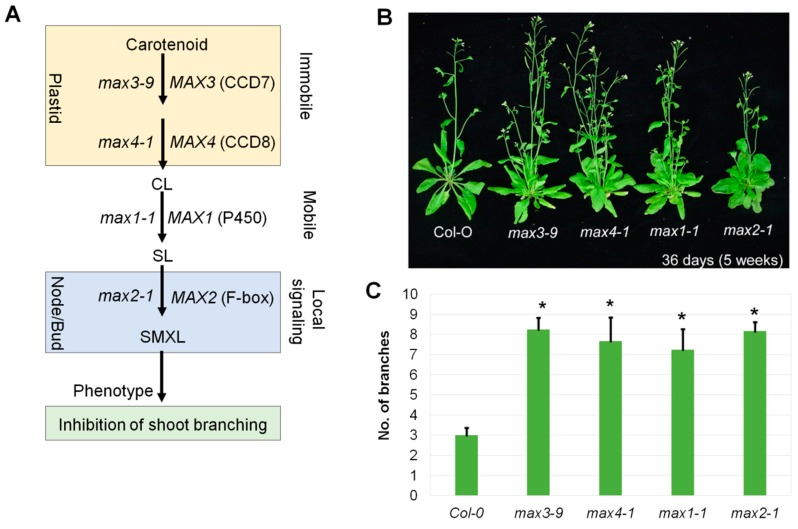
Strigolactone biosynthesis pathway and perception. (**A**) Strigolactone biosynthesis, signaling perception pathway and mutants identified by genetic methods in this pathway. (**B**) The *max* mutants with their branched phenotypes. (**C**) Number of branches per *max* mutant compared with the wild-type. Bars indicate standard error of five replicates. The asterisk sign (∗) indicates the significant difference between *max* mutants and Col-0 at *p* < 0.05 (Student’s *t*-test).

The SL signaling pathway is one of the important pathways that determine branch phenotype along with other phenotypes in plants (Figure 1A). Genes involved in SL signaling were identified based on more branching phenotypes in Arabidopsis, pea, and rice mutant [22,25]. In Arabidopsis, four *MAX* genes are involved in the SL pathway. These genes translate into functional protein enzymes, such as CCD7 (*MAX3*), CCD8 (*MAX4*), P450 (*MAX1*), and F-box (*MAX2*) [16,34,35,39]. Their mutants (*max3-9*, *max4-1*, *max1-1*, and *max2-1*) have more branch phenotypes (Figure 1B,C), as shown in previous studies [16,34,37,39]. The high branched phenotype of the *max* mutant can be rescued by application of an extremal analogue of SL (synthetic strigolactone), GR24, except for the *max2* phenotype [10,11]. DWARF27 is an iron-containing protein that is required for the biosynthesis of SLs, and it regulates rice tiller bud outgrowth [40]. It also catalyzes all *trans*/9-*cis* to isomerization of β-carotene [31,41]. CCD7 cleaves 9-*cis*-β-carotene into the volatile compounds β-ionone and 9-cis-β-apo-10′-carotenal [31,42]. CCD8 converts 9-*cis*-β-apo-10′-carotenal into carlactone that resembles SLs in the number of C-atoms [31]. The Arabidopsis MAX1 enzyme, P450 converts carlactone into intermediate methylated carlactonoic acid that binds to the SL receptor α/β-hydrolase D14 [43]. Methylated carlactonoic acid is hydroxylated by lateral branching oxidoreductase (LBO) into a yet unidentified compound [44]. F-box protein MAX2 interacts with α/β-hydrolase D14 and mediates SL signaling by directing its targeted proteins, such as SUPPRESSOR OF MORE AXILLARY GROWTH2-LIKE6 (SMXL6), SMXL7, and SMXL8 to proteasomal degradation [13,45]. It would be interesting to see the common differentially expressed genes in *max* mutants to understand the downstream genes of the SL signaling pathway.

### 2.2. Microarray Analysis Revealed Differentially Expressed Genes (DEGs) in max Mutants and the Role of Those Genes in the Biological Process

**Figure 2:**
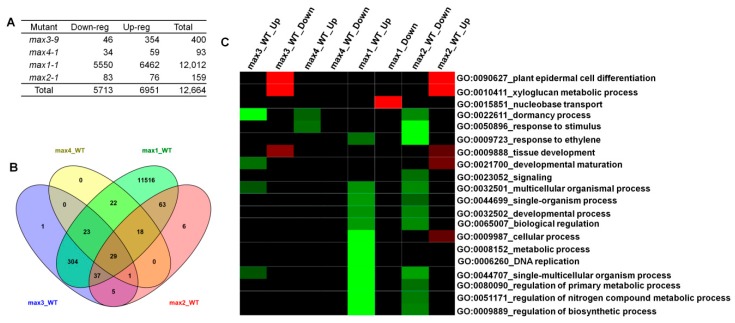
Microarray analysis of the gene expression profiles in *max* mutants compared with wild-type. (**A**) Significantly changed genes in *max* mutants compared with wild-type. (**B**) Venn diagram for several genes that changed expressed levels in mutants compared with those of wild-type control. (**C**) Heatmap of differentially expressed Gene Ontology (GO) in all four *max* mutants. Panel on the right side of the heat map shows the GO identity and its role in various biological processes. Red boxes in the heat map show up-regulated GO, green boxes show down-regulated GO, and black boxes show unchanged GO expression in four *max* mutants.

Transcriptomic analysis was performed on four *max* mutant plants and compared with wild-type plants, using the Roche NimbleGen microarray system [46]. The experiment was performed on each independent biological sample. Preprocessed data were exported to the Limma package in R language [47]. An adjusted P-value was estimated using empirical Bayesian statistics implemented using eBayes. Significant DEGs were identified as those with log2 FC (fold-change) > 1 for up-regulation and log2 FC (fold-change) < –1 for down-regulation with an adjusted *p*-value < 0.05. Five comparisons were performed with: wild type-control versus wild type-control to normalize the expression within the control group; *max1*, *max2*, *max3*, and *max4* mutants versus wild-type to determine differentially expressed transcripts in the *max* mutants (Figure S1). Among the 39,042 genes identified in the NimbleGen array, 12,664 genes were differentially expressed (with *p*-value < 0.05) in the *max* mutants versus wild-type control comparison, corresponding to 34.1% of the total genes (Figure S1; Table S2). It is comprised of 6951 (18.7%) up-regulated genes and 5713 (15.4%) down-regulated genes (Figure 2A). In the *max3-9* mutant, a total of 400 genes were differentially expressed, and among those genes, 46 were down-regulated, and 354 were up-regulated. In the *max4-1* mutant, a total of 93 genes were differentially expressed, and among those genes, 34 were down-regulated, and 59 were up-regulated. Surprisingly in the *max1-1* mutant, total 12,012 genes were differentially expressed, and among those genes 5550 were down-regulated, and 6462 were up-regulated (Figure 2A). The reason for a large number of genes differentially expressed in *max1* mutant might be due to the accumulation of a large amount of carlactone (CL) [48]. Since CL acts as a signal molecule, it might invoke the expression of many genes [43]. Finally, in the *max2-1* mutant, a total of 159 genes were differentially expressed, and among those genes, 83 were down-regulated, and 76 were up-regulated (Figure 2A). Venn diagram explains commonly expressed genes between mutant lines (Figure 2B). Twenty-nine genes were commonly expressed in all four mutants (Table S3). Sixty-three genes were commonly expressed between *max1* and *max2* mutants (Table S4). Five genes were commonly expressed in the *max3* and *max2* mutants (Table S5). Three-hundred-and-four genes were commonly expressed in the *max3* and *max1* mutants (Table S6). Twenty-two genes were commonly expressed in the *max4* and *max1* mutants (Table S7). In *max3*, *max1*, and *max2* mutants, the number of genes differentially expressed were 1, 11, 516, and 5, respectively (Tables S8–S10). The *max4* mutant did not show any *max4*-specific differentially expressed genes (Figure 2B). 

Differentially down- or up-regulated genes enriched in GO terms were functionally classified in the *max* mutants (Figure 2C). Terms containing up-regulated genes in *max3* were GO:0090627 (plant epidermal cell differentiation) and GO:0010411 (xyloglucan metabolic process). GO:0022611 (dormancy process) displayed significantly down-regulated genes. Marginally down-regulated genes were found in *max4* and are associated with GO:0022611 (dormancy process) and GO:0050896 (response to stimulus). In *max1*, GO:0015851 (nucleobase transport) was significantly enriched while many genes were significantly down-regulated in GO terms. In *max2*, enrichment was the same as *max3,* and many biological terms were down-regulated, but the degree of extent was less than those of *max1*.

### 2.3. Strigolactone Metabolism-Related Genes Showed Differential Expression Pattern in the max Mutant

**Figure 3:**
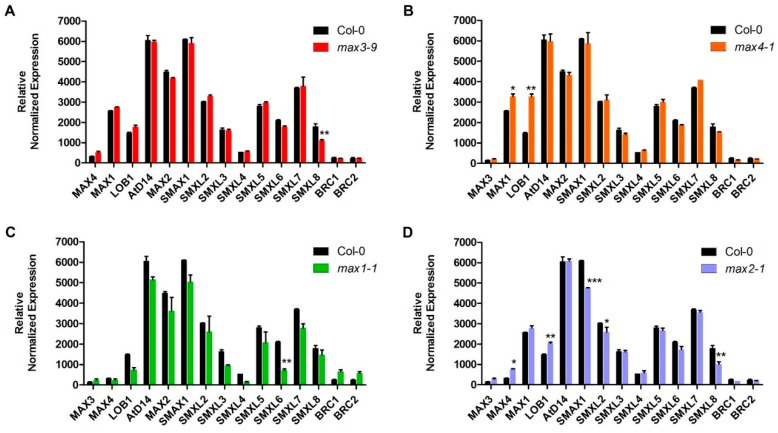
The transcript level of genes involved in strigolactone biosynthesis and signaling pathway in four *max* mutants. (**A**–**D**) Relative expression of SL metabolism-related genes in *max3-9*, *max4-1*, *max1-1*, and *max2-1* mutant respectively compared with wild-type based on microarray results. Leaf of the mature plant was used to isolate RNA. Each data point represents the mean (±SE) from 2 different plants. The star indicates a significant difference between mutant and wild-type. * *p* < 0.05, ** *p* < 0.05, *** *p* < 0.001 (Two-way ANOVA test).

A close look at the expression levels of known SLs biosynthesis and signaling related genes in *max* mutant plants revealed a more significant role of MAX2 (Figure 3). The expression of most of the SLs signaling related genes were induced or repressed in the *max2* mutant (Figure 3D). There was some exception in other *max* mutants where few SL signaling-related genes showed differential expression patterns, such as repression of *SUPPRESSOR OF MAX2 LIKE 8* (*SMXL8*) in the *max3* mutant, increased expression of *MAX1* and *LOB1* in the *max4* mutant, and repression of *SUPPRESSOR OF MAX2 LIKE 6* (*SMXL6*) in the *max1* mutant. However, it was the *max2* mutant that showed significant differential expression of the SLs signaling related genes. *LATERAL ORGAN BOUNDARIES* (*LOB1*) negatively regulates brassinosteroid accumulation to limit growth in organ boundaries [49]. *LOB1* expression was up-regulated in all *max* mutants except *max1*. It might be due to the absence of *MAX* genes; there is no controlled regulation of *LOB1* for the growth limit. In our analysis, *SUPPRESSOR OF MAX2 1 (SMAX1), SMAX-LIKE2 (SMXL2)* and *SMAX-LIKE8 (SMXL8)* expressions were significantly down-regulated in the *max2* mutant, which makes sense since there is no MAX2 in the mutant and SMAXLs may not be able to be regulated by MAX2. This result is in correlation with the previously published result where increased branching phenotype of *max2* rescued in the line where *SMAX1*, *SMXL2,* and *SMXL8* were constitutively targeted by artificial microRNAs [50].

### 2.4. Expression Analysis Revealed Candidate Genes Involved in Downstream SL Pathway

**Figure 4:**
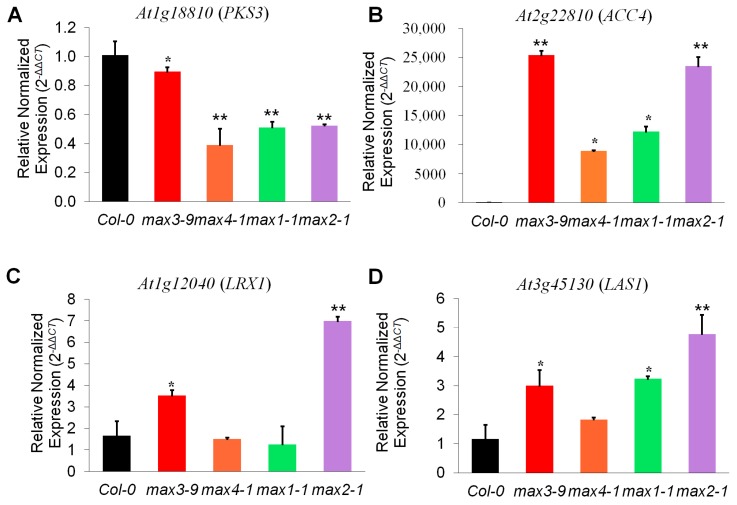
Differentially expressed genes in *max2* mutants. (**A**–**D**) Relative normalized gene expression compared to wild-type in the *max* mutant. Error bars represent standard error of three replicates. Leaf of the mature plant was used to isolate RNA. The star indicates a significant difference between mutant and wild-type. * *p* < 0.05, ** *p* < 0.01 (Student’s *t*-test).

**Figure 5:**
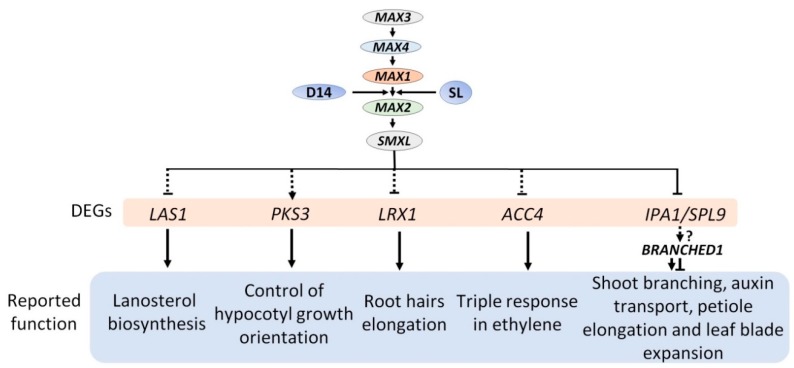
Proposed SL signaling pathways. DEGs means differential expressed genes. Black arrows represent up-regulated genes, dotted lines, and question mark (?) represent unreported genes.

We focused on the highly differentially regulated genes in the *max* mutant, and we narrowed down to four genes. For the validation of microarray data, real-time PCR analysis was performed, and specific primers were designed for it (Table S1). Three genes, namely, *1-AMINOCYCLOPROPANE-1-CARBOXYLATE SYNTHASE 4* (*ACC4*), *LEUCINE-RICH REPEAT/EXTENSIN 1* (*LRX1*), and *LANOSTEROL SYNTHASE 1* (*LAS1*) gene, showed induced expression, whereas one gene, *PROTEIN KINASE 3* (*PKS3*) expression was down-regulated in the *max* mutant (Figure 4). These genes reportedly played a different set of role such as the expression of the *ACC4*, a key regulatory enzyme in the biosynthesis of the plant hormone ethylene repressed by ABA INSENSITIVE4 (ABI4) by directly binding to their promoters [51]. Additionally, *atacc4* showed reduced branching, increased plant height, and hypocotyl length, which is precisely antagonistic to the phenotype of *max* mutant [52,53,54]. It also explains the cooperation between strigolactone signaling and ACC4. For root hair elongation in Arabidopsis, it is also reported that ethylene interacts with strigolactone [15]. PKS3 is a phytochrome kinase substrate-like protein related to control of hypocotyl growth orientation [55], and *max2* hypocotyls were also more elongated compared with wild-type [56]. It indicated that to control hypocotyl growth, and there is some working mechanism going on between SL signaling and PKS3. Similarly, *lrx1* have short root hair and might be regulated by MAX2 since its expression is high in *max2* [57]. *LAS1* is involved in the lanosterol biosynthesis, but *las1* mutant did not display any phenotype [58,59]. There is no report of any relation between *LAS1* and MAX. It is the first time we have reported that expression of *LAS1* increase in *max* mutant and further study needed to confirm if *LAS1* is functionally involved in SL signaling. Based on our findings, we proposed a MAX dependent model (Figure 5).

## 3. Materials and Methods

### 3.1. Plant Materials and Growth Conditions

*Arabidopsis thaliana* used in this study included the wild-type Columbia (Col-0) ecotype and mutants: *max3-9* [34], *max4-1* [16], *max1-1* and *max2-1* [37]. Arabidopsis seeds were sterilized and vernalized at 4 °C for 3 d in the dark. It is germinated on 1/2 Murashige and Skoog (MS) medium containing 0.7% (*w*/*v*) agar and 1.0% (*w*/*v*) sucrose. For phenotypical observations, 12-d-old seedlings transferred to soil in small pots. Arabidopsis plants were grown for five weeks under a 16-h-light/8-h-dark cycle with a light intensity of 60 to 80 µE m^–2^ s^–1^ at ~21 °C [60].

### 3.2. Microarray Hybridization and Data Analysis

The 135k microarray was prepared from 39,042 genes (TAIR v.9). Four, sixty-nt long probes per gene were outlined. NimbleGen Inc. (Madison, WI, USA) manufactured the microarray (http://www.nimblegen.com/). Random 40,000 GC probes were included to assist with overlaying the grid on the image for the monitoring of the hybridization efficiency and four corner fiducial controls (225). We repeated the experiment 3 times and independently prepared total RNA from the leaf of the mature plant for the reproducibility of microarray analysis [61]. Ten micrograms of Cy3-labeled target DNA fragments were used for microarray hybridization. For hybridization, an MAUI chamber (Biomicro, Salt Lake, UT, USA) was used at 42 °C for 16–18 h. The data were processed and normalized by cubic spline normalization using quantiles to adjust signal variations between chips [62]. For producing call files, a probe-level summarization by Robust Multi-Chip Analysis (RMA) using a median polish algorithm was implemented in NimbleScan. Limma package in the R computing environment was used for multiple analysis [47]. Genes having p-value below 0.05 or false discovery were collected. Multivariate statistical tests such as clustering, multidimensional scaling, and principal component analysis were performed by Acuity 3.1 (Axon Instruments, Union City, CA, USA) [63]. 

Biological term enrichment was assessed using GoMiner^TM^ [64], as described previously [65]. The microarray contained 39,042 genes; these genes were used as the total gene set in GoMiner. Among the 39,042 genes, 545 were associated with biological processes, 96 with molecular functions, and 68 with cellular components. Top terms in each clad were selected to reduce redundancy, and hierarchical clustering was performed with the Bioconductor R package 3.9 (http://master.bioconductor.org/). GO terms with FDR values less than 0.05 were considered as significant. For color presentation, up-regulated terms were scaled 0.5 to 5 and indicated as red code in Figure 2C. Similarly, down-regulated terms were scaled –0.5 to –5 and indicated as blue code [66]. Other non-significant FDR values were transformed to 0 with Perl scripting language [65]. 

### 3.3. Quantitative Real-Time RT-PCR Analysis 

Total RNA from plant leaf was isolated using the RNAeasy Mini Kit—Qiagen (Hilden, Germany). Isolated RNA was treated with DNAse I (Takara Bio, Shiga, Japan) to avoid contamination with genomic DNA. RNA was reverse transcribed using a Reverse Transcription kit (Takara Bio, Shiga, Japan). Two micrograms of RNA were used for cDNA synthesis according to manual instructions. Ten-fold diluted cDNA was used as a template for real-time quantitative PCR using SYBR Mix (Roche Diagnostics). Gene-specific forward and reverse primers were used, five µM of each. Gene-specific qRT-PCR primers were designed using NCBI primer blast tool and synthesized (Bioneer, Daejeon, Korea). The primers are listed in Supplementary Table S1. The genes were amplified using a StepOnePlus Real-Time PCR System (Applied Biosystems, Foster City, CA, USA). For normalizing expression levels of a constitutively expressed gene, *EIF4A1* was used as a reference gene. The thermal profile used consisted of an initial denaturation step at 95 °C for 5 min, followed by 40 cycles of 95 °C for 10 s, annealing for 15 s, and 72 °C for 15 s followed by one cycle of 95 °C for 10 s.

## 4. Conclusions

SL signaling-related knowledge has been rapidly increasing. Recently, a study indicated that a transcription factor, *IDEAL PLANT ARCHITECTURE1* (*IPA1*), is directly involved to SL signaling and regulates shoot branching [67] (Figure 5). However, this pathway still lacks basic answer, particularly, the molecular mechanism which identifies its downstream genes, and those genes are the direct or indirect target. Our study identifies DEGs that may act downstream of the SL signaling network for various functions.

## Supplementary Materials

The following are available online at https://www.mdpi.com/2223-7747/8/9/352/s1, Figure S1: Comparison of signal distribution between biological repeats of samples, Figure S2: Hierarchical clustering of significantly changed genes of expression *max* mutants compared with wild-type and the log2 ratio used for normalizing the expression difference, Table S1: List of primers used in this study for qRT-PCR analysis, Table S2: The list of the genes that significantly increased or decreased in *max* mutants compared to wild-type, Table S3: Commonly expresses genes in *max3*, *max4*, *max1*, and *max2*, Table S4: Commonly expressed genes in *max1* and *max2*, Table S5: Commonly expressed genes in *max3* and *max2*, Table S6: Commonly expressed genes in *max3* and *max1*, Table S7: Commonly expressed genes in *max4* and *max1*, Table S8: A list of *max3*-specific changed gene, Table S9: A list of *max1*-specific changed genes, Table S10: A list of *max2*-specific changed genes.

## Abbreviations

CCD7	CAROTENOID CLEAVAGE DIOXYGENASE 7
CCD8	CAROTENOID CLEAVAGE DIOXYGENASE 8
SL	STRIGOLACTONE
IPA1	IDEAL PLANT ARCHITECTURE 1
ABI4	ABA INSENSITIVE 4
ACC4	AMINOCYCLOPROPANE-1-CARBOXYLATE SYNTHASE 4
LRX1	LEUCINE-RICH REPEAT/EXTENSIN 1
LAS1	LANOSTEROL SYNTHASE 1
PKS3	PROTEIN KINASE 3
GO	GENE ONTOLOGY

## References

[1] Xie X., Yoneyama K., Yoneyama K. (2010). The strigolactone story. Annu. Rev. Phytopathol..

[2] Cook C.E., Whichard L.P., Turner B., Wall M.E., Egley G.H. (1966). Germination of Witchweed (Striga lutea Lour.): Isolation and Properties of a Potent Stimulant. Science.

[3] Bouwmeester H.J., Matusova R., Zhongkui S., Beale M.H. (2003). Secondary metabolite signalling in host-parasitic plant interactions. Curr. Opin. Plant Biol..

[4] Cheng X., Ruyter-Spira C., Bouwmeester H. (2013). The interaction between strigolactones and other plant hormones in the regulation of plant development. Front. Plant Sci..

[5] Gutjahr C., Parniske M. (2013). Cell and developmental biology of arbuscular mycorrhiza symbiosis. Annu. Rev. Cell Dev. Biol..

[6] Akiyama K., Matsuzaki K., Hayashi H. (2005). Plant sesquiterpenes induce hyphal branching in arbuscular mycorrhizal fungi. Nature.

[7] Besserer A., Becard G., Jauneau A., Roux C., Sejalon-Delmas N. (2008). GR24, a synthetic analog of strigolactones, stimulates the mitosis and growth of the arbuscular mycorrhizal fungus Gigaspora rosea by boosting its energy metabolism. Plant Physiol..

[8] Kumar M., Pandya-Kumar N., Kapulnik Y., Koltai H. (2015). Strigolactone signaling in root development and phosphate starvation. Plant Signal. Behav..

[9] Yoneyama K., Xie X.N., Kim H.I., Kisugi T., Nomura T., Sekimoto H., Yokota T., Yoneyama K. (2012). How do nitrogen and phosphorus deficiencies affect strigolactone production and exudation?. Planta.

[10] Gomez-Roldan V., Fermas S., Brewer P.B., Puech-Pages V., Dun E.A., Pillot J.P., Letisse F., Matusova R., Danoun S., Portais J.C. (2008). Strigolactone inhibition of shoot branching. Nature.

[11] Umehara M., Hanada A., Yoshida S., Akiyama K., Arite T., Takeda-Kamiya N., Magome H., Kamiya Y., Shirasu K., Yoneyama K. (2008). Inhibition of shoot branching by new terpenoid plant hormones. Nature.

[12] Agusti J., Herold S., Schwarz M., Sanchez P., Ljung K., Dun E.A., Brewer P.B., Beveridge C.A., Sieberer T., Sehr E.M. (2011). Strigolactone signaling is required for auxin-dependent stimulation of secondary growth in plants. Proc. Natl. Acad. Sci. USA.

[13] Hamiaux C., Drummond R.S.M., Janssen B.J., Ledger S.E., Cooney J.M., Newcomb R.D., Snowden K.C. (2012). DAD2 Is an alpha/beta Hydrolase Likely to Be Involved in the Perception of the Plant Branching Hormone, Strigolactone. Curr. Biol..

[14] Kapulnik Y., Delaux P.M., Resnick N., Mayzlish-Gati E., Wininger S., Bhattacharya C., Sejalon-Delmas N., Combier J.P., Becard G., Belausov E. (2011). Strigolactones affect lateral root formation and root-hair elongation in Arabidopsis. Planta.

[15] Kapulnik Y., Resnick N., Mayzlish-Gati E., Kaplan Y., Wininger S., Hershenhorn J., Koltai H. (2011). Strigolactones interact with ethylene and auxin in regulating root-hair elongation in Arabidopsis. J. Exp. Bot..

[16] Sorefan K., Booker J., Haurogne K., Goussot M., Bainbridge K., Foo E., Chatfield S., Ward S., Beveridge C., Rameau C. (2003). MAX4 and RMS1 are orthologous dioxygenase-like genes that regulate shoot branching in Arabidopsis and pea. Genes Dev..

[17] Li S.W., Xue L.G., Xu S.J., Feng H.Y., An L.Z. (2009). Mediators, Genes and Signaling in Adventitious Rooting. Bot. Rev..

[18] Rasmussen A., Mason M.G., De Cuyper C., Brewer P.B., Herold S., Agusti J., Geelen D., Greb T., Goormachtig S., Beeckman T. (2012). Strigolactones Suppress Adventitious Rooting in Arabidopsis and Pea. Plant Physiol..

[19] Ruyter-Spira C., Kohlen W., Charnikhova T., van Zeijl A., van Bezouwen L., de Ruijter N., Cardoso C., Lopez-Raez J.A., Matusova R., Bours R. (2011). Physiological Effects of the Synthetic Strigolactone Analog GR24 on Root System Architecture in Arabidopsis: Another Belowground Role for Strigolactones?. Plant Physiol..

[20] Snowden K.C., Simkin A.J., Janssen B.J., Templeton K.R., Loucas H.M., Simons J.L., Karunairetnam S., Gleave A.P., Clark D.G., Klee H.J. (2005). The Decreased apical dominance1/Petunia hybrida CAROTENOID CLEAVAGE DIOXYGENASE8 gene affects branch production and plays a role in leaf senescence, root growth, and flower development. Plant Cell.

[21] Brewer P.B., Koltai H., Beveridge C.A. (2013). Diverse Roles of Strigolactones in Plant Development. Mol. Plant.

[22] Seto Y., Yamaguchi S. (2014). Strigolactone biosynthesis and perception. Curr. Opin. Plant Biol..

[23] Kapulnik Y., Koltai H. (2014). Strigolactone Involvement in Root Development, Response to Abiotic Stress, and Interactions with the Biotic Soil Environment. Plant Physiol..

[24] Waldie T., McCulloch H., Leyser O. (2014). Strigolactones and the control of plant development: Lessons from shoot branching. Plant J..

[25] Al-Babili S., Bouwmeester H.J. (2015). Strigolactones, a Novel Carotenoid-Derived Plant Hormone. Annu. Rev. Plant Biol..

[26] Decker E.L., Alder A., Hunn S., Ferguson J., Lehtonen M.T., Scheler B., Kerres K.L., Wiedemann G., Safavi-Rizi V., Nordzieke S. (2017). Strigolactone biosynthesis is evolutionarily conserved, regulated by phosphate starvation and contributes to resistance against phytopathogenic fungi in a moss, Physcomitrella patens. New Phytol..

[27] Fraser P.D., Bramley P.M. (2004). The biosynthesis and nutritional uses of carotenoids. Prog. Lipid Res..

[28] Moise A.R., Al-Babili S., Wurtzel E.T. (2014). Mechanistic Aspects of Carotenoid Biosynthesis. Chem. Rev..

[29] Nisar N., Li L., Lu S., Khin N.C., Pogson B.J. (2015). Carotenoid Metabolism in Plants. Mol. Plant.

[30] Walter M.H., Strack D. (2011). Carotenoids and their cleavage products: Biosynthesis and functions. Nat. Prod. Rep..

[31] Alder A., Jamil M., Marzorati M., Bruno M., Vermathen M., Bigler P., Ghisla S., Bouwmeester H., Beyer P., Al-Babili S. (2012). The Path from beta-Carotene to Carlactone, a Strigolactone-Like Plant Hormone. Science.

[32] Braun N., de Saint Germain A., Pillot J.P., Boutet-Mercey S., Dalmais M., Antoniadi I., Li X., Maia-Grondard A., Le Signor C., Bouteiller N. (2012). The Pea TCP Transcription Factor PsBRC1 Acts Downstream of Strigolactones to Control Shoot Branching. Plant Physiol..

[33] Turnbull C.G.N., Booker J.P., Leyser H.M.O. (2002). Micrografting techniques for testing long-distance signalling in Arabidopsis. Plant J..

[34] Booker J., Auldridge M., Wills S., McCarty D., Klee H., Leyser O. (2004). MAX3/CCD7 is a carotenoid cleavage dioxygenase required for the synthesis of a novel plant signaling molecule. Curr. Biol..

[35] Booker J., Sieberer T., Wright W., Williamson L., Willett B., Stirnberg P., Turnbull C., Srinivasan M., Goddard P., Leyser O. (2005). MAX1 encodes a cytochrome P450 family member that acts downstream of MAX3/4 to produce a carotenoid-derived branch-inhibiting hormone. Dev. Cell.

[36] Auldridge M.E., Block A., Vogel J.T., Dabney-Smith C., Mila I., Bouzayen M., Magallanes-Lundback M., DellaPenna D., McCarty D.R., Klee H.J. (2006). Characterization of three members of the Arabidopsis carotenoid cleavage dioxygenase family demonstrates the divergent roles of this multifunctional enzyme family. Plant J..

[37] Stirnberg P., van de Sande K., Leyser H.M.O. (2002). MAX1 and MAX2 control shoot lateral branching in Arabidopsis. Development.

[38] Stirnberg P., Furner I.J., Leyser H.M.O. (2007). MAX2 participates in an SCF complex which acts locally at the node to suppress shoot branching. Plant J..

[39] Ul Haq B., Ahmad M.Z., Rehman N.U., Wang J.J., Li P.H., Li D.Q., Zhao J. (2017). Functional characterization of soybean strigolactone biosynthesis and signaling genes in Arabidopsis MAX mutants and GmMAX3 in soybean nodulation. BMC Plant Biol..

[40] Lin H., Wang R.X., Qian Q., Yan M.X., Meng X.B., Fu Z.M., Yan C.Y., Jiang B., Su Z., Li J.Y. (2009). DWARF27, an Iron-Containing Protein Required for the Biosynthesis of Strigolactones, Regulates Rice Tiller Bud Outgrowth. Plant Cell.

[41] Bruno M., Al-Babili S. (2016). On the substrate specificity of the rice strigolactone biosynthesis enzyme DWARF27. Planta.

[42] Bruno M., Hofmann M., Vermathen M., Alder A., Beyer P., Al-Babili S. (2014). On the substrate- and stereospecificity of the plant carotenoid cleavage dioxygenase 7. FEBS Lett..

[43] Abe S., Sado A., Tanaka K., Kisugi T., Asami K., Ota S., Kim H., Yoneyama K., Xie X., Ohnishi T. (2014). Carlactone is converted to carlactonoic acid by MAX1 in Arabidopsis and its methyl ester can directly interact with AtD14 in vitro. Proc. Natl. Acad. Sci. USA.

[44] Brewer P.B., Yoneyama K., Filardo F., Meyers E., Scaffidi A., Frickey T., Akiyama K., Seto Y., Dun E.A., Cremer J.E. (2016). LATERAL BRANCHING OXIDOREDUCTASE acts in the final stages of strigolactone biosynthesis in Arabidopsis. Proc. Natl. Acad. Sci. USA.

[45] Wang L., Wang B., Jiang L., Liu X., Li X.L., Lu Z.F., Meng X.B., Wang Y.H., Smith S.M., Li J.Y. (2015). Strigolactone Signaling in Arabidopsis Regulates Shoot Development by Targeting D53-Like SMXL Repressor Proteins for Ubiquitination and Degradation. Plant Cell.

[46] Dalma-Weiszhausz D.D., Warrington J., Tanimoto E.Y., Miyada C.G. (2006). The affymetrix GeneChip platform: An overview. Methods Enzym..

[47] Smyth G.K. (2004). Linear models and empirical bayes methods for assessing differential expression in microarray experiments. Stat. Appl. Genet. Mol. Biol..

[48] Seto Y., Sado A., Asami K., Hanada A., Umehara M., Akiyama K., Yamaguchi S. (2014). Carlactone is an endogenous biosynthetic precursor for strigolactones. Proc. Natl. Acad. Sci. USA.

[49] Bell E.M., Lin W.C., Husbands A.Y., Yu L.F., Jaganatha V., Jablonska B., Mangeon A., Neff M.M., Girke T., Springer P.S. (2012). Arabidopsis LATERAL ORGAN BOUNDARIES negatively regulates brassinosteroid accumulation to limit growth in organ boundaries. Proc. Natl. Acad. Sci. USA.

[50] Soundappan I., Bennett T., Morffy N., Liang Y.Y., Stang J.P., Abbas A., Leyser O., Nelson D.C. (2015). SMAX1-LIKE/D53 Family Members Enable Distinct MAX2-Dependent Responses to Strigolactones and Karrikins in Arabidopsis. Plant Cell.

[51] Dong Z.J., Yu Y.W., Li S.H., Wang J., Tang S.J., Huang R.F. (2016). Abscisic Acid Antagonizes Ethylene Production through the ABI4-Mediated Transcriptional Repression of ACS4 and ACS8 in Arabidopsis. Mol. Plant.

[52] Tsuchisaka A., Yu G.X., Jin H.L., Alonso J.M., Ecker J.R., Zhang X.M., Gao S., Theologis A. (2009). A Combinatorial Interplay Among the 1-Aminocyclopropane-1-Carboxylate Isoforms Regulates Ethylene Biosynthesis in *Arabidopsis thaliana*. Genetics.

[53] Zhao Q.P., Wang X.N., Li N.N., Zhu Z.Y., Mu S.C., Zhao X., Zhang X. (2018). Functional Analysis of MAX2 in Phototropins-Mediated Cotyledon Flattening in Arabidopsis. Front. Plant Sci..

[54] Jia K.P., Luo Q., He S.B., Lu X.D., Yang H.Q. (2014). Strigolactone-Regulated Hypocotyl Elongation Is Dependent on Cryptochrome and Phytochrome Signaling Pathways in Arabidopsis. Mol. Plant.

[55] Demarsy E., Schepens I., Okajima K., Hersch M., Bergmann S., Christie J., Shimazaki K., Tokutomi S., Fankhauser C. (2012). Phytochrome Kinase Substrate 4 is phosphorylated by the phototropin 1 photoreceptor. EMBO J..

[56] Nelson D.C., Scaffidi A., Dun E.A., Waters M.T., Flematti G.R., Dixon K.W., Beveridge C.A., Ghisalberti E.L., Smith S.M. (2011). F-box protein MAX2 has dual roles in karrikin and strigolactone signaling in *Arabidopsis thaliana*. Proc. Natl. Acad. Sci. USA.

[57] Diet A., Link B., Seifert G.J., Schellenberg B., Wagner U., Pauly M., Reiter W.D., Ringli C. (2006). The Arabidopsis root hair cell wall formation mutant lrx1 is suppressed by mutations in the RHM1 gene encoding a UDP-L-rhamnose synthase. Plant Cell.

[58] Kolesnikova M.D., Xiong Q.B., Lodeiro S., Hua L., Matsuda S.P.T. (2006). Lanosterol biosynthesis in plants. Arch. Biochem. Biophys..

[59] Ohyama K., Suzuki M., Kikuchi J., Saito K., Muranaka T. (2009). Dual biosynthetic pathways to phytosterol via cycloartenol and lanosterol in Arabidopsis. Proc. Natl. Acad. Sci. USA.

[60] Dai Y., Wang H., Li B., Huang J., Liu X., Zhou Y., Mou Z., Li J. (2006). Increased expression of MAP KINASE KINASE7 causes deficiency in polar auxin transport and leads to plant architectural abnormality in Arabidopsis. Plant Cell.

[61] Cho W.K., Yu J., Lee K.M., Son M., Min K., Lee Y.W., Kim K.H. (2012). Genome-wide expression profiling shows transcriptional reprogramming in Fusarium graminearum by Fusarium graminearum virus 1-DK21 infection. BMC Genom..

[62] Workman C., Jensen L.J., Jarmer H., Berka R., Gautier L., Nielser H.B., Saxild H.H., Nielsen C., Brunak S., Knudsen S. (2002). A new non-linear normalization method for reducing variability in DNA microarray experiments. Genome Biol..

[63] Minh-Thu P.T., Hwang D.J., Jeon J.S., Nahm B.H., Kim Y.K. (2013). Transcriptome analysis of leaf and root of rice seedling to acute dehydration. Rice.

[64] Zeeberg B.R., Feng W.M., Wang G., Wang M.D., Fojo A.T., Sunshine M., Narasimhan S., Kane D.W., Reinhold W.C., Lababidi S. (2003). GoMiner: A resource for biological interpretation of genomic and proteomic data. Genome Biol..

[65] Chae S., Kim J.S., Jun K.M., Lee S.B., Kim M.S., Nahm B.H., Kim Y.K. (2017). Analysis of Genes with Alternatively Spliced Transcripts in the Leaf, Root, Panicle and Seed of Rice Using a Long Oligomer Microarray and RNA-Seq. Mol. Cells.

[66] Meur N.L., Gentleman R. (2012). Analyzing biological data using R: Methods for graphs and networks. Methods Mol Biol..

[67] Kerr S.C., Beveridge C.A. (2017). IPA1: A direct target of SL signaling. Cell Res..

